# A Novel Portable Gamma Radiation Sensor Based on a Monolithic Lutetium-Yttrium Oxyorthosilicate Ring

**DOI:** 10.3390/s21103376

**Published:** 2021-05-12

**Authors:** Xi Zhang, Qiangqiang Xie, Siwei Xie, Xin Yu, Jianfeng Xu, Qiyu Peng

**Affiliations:** 1 The School of Mechanical Science and Engineering, Huazhong University of Science and Technology, Wuhan 430074, China; xizhang@hust.edu.cn (X.Z.); xieqiang@hust.edu.cn (Q.X.); M202070480@hust.edu.cn (X.Y.); 2 Shenzhen Bay Laboratory, The Institute of Biomedical Engineering, Shenzhen 518132, China; xiesw@szbl.ac.cn (S.X.); qpeng@lbl.gov (Q.P.)

**Keywords:** gamma radiation, monolithic LYSO ring, Monte Carlo simulation, position decoding

## Abstract

Portable radiation detectors are widely used in environmental radiation detection and medical imaging due to their portability feature, high detection efficiency, and large field of view. Lutetium-yttrium oxyorthosilicate (LYSO) is a widely used scintillator in gamma radiation detection. However, the structure and the arrangement of scintillators limit the sensitivity and detection accuracy of these radiation detectors. In this study, a novel portable sensor based on a monolithic LYSO ring was developed for the detection of environmental radiation through simulation, followed by construction and assessments. Monte Carlo simulations were utilized to prove the detection of gamma rays at 511 keV by the developed sensor. The simulations data, including energy resolutions, decoding errors, and sensitivity, showed good potential for the detection of gamma rays by the as-obtained sensor. The experimental results using the VA method revealed decoding errors in the energy window width of 50 keV less than 2°. The average error was estimated at 0.67°, a sufficient value for the detection of gamma radiation. In sum, the proposed radiation sensor appears promising for the construction of high-performance radiation detectors and systems.

## 1. Introduction

Radiation detectors are suitable for use in environmental radiation detection and medical imaging due to their portability features coupled with high detection efficiencies and large field of view [1,2,3]. In recent years, portable gamma camera systems have attracted attention not only in radio-guided surgery [4,5], but also in radiation security to minimize radiation exposure and detect radiation sources [6,7,8].

Recently, the development of crystal materials and photoelectric sensing technologies has completely changed the key components of portable gamma radiation detectors [9,10,11]. Lutetium-yttrium oxyorthosilicate (LYSO) is a scintillation crystal with efficient radiation detection characteristics, such as short decay time and high light yield [12]. LYSO has been used as an alternative to bismuth germanate (BGO) and has become the most popular scintillator in nuclear imaging systems, such as single-photon emission computed tomography (SPECT) and positron emission tomography (PET) [13,14]. For photon detection, photomultiplier tube (PMT) instruments are still the most popular photon sensors used in the last few decades. However, PMTs are limited by their large size and poor ability to resist magnetic field interference. Silicon photomultiplier (SiPM)-based sensors are being gradually used as a replacement for PMTs thanks to their elevated detection efficiencies, low dark currents, high amplitude, and anti-magnetic abilities [15,16].

Gate is a piece of Monte Carlo simulation software popular in the field of nuclear medical imaging [17,18]. Users can easily simulate physical processes associated with high-energy particles and rays since GATE provides various PET and SPECT system standard models. GATE is also useful for optical simulations and the interaction of photons with the crystal surface [19,20]. Two optical models exist to simulate the propagation on the target boundary: (i) look-up-table (LUT) Davis model and (ii) unified model. GATE can be used in optical simulation to properly define material properties, such as characteristics associated with the crystal material and reflection, as well as the reflection model.

Traditional gamma-ray detectors based on scintillators are often manufactured using a pixeled crystal array [9,21,22]. However, this design is limited by the degradation of spatial resolution when gamma rays enter obliquely. Moreover, only gamma rays coming from the end face could be used to locate the gamma source. This greatly influences the sensitivity of the gamma camera. To overcome these issues, different types of collimators are used to improve the field of view (FOV) and accuracy of the gamma camera [7,18,23]. These include parallel holes, holes, and converging and diverging collimators. Nevertheless, the use of collimators cannot solve problems linked to high sensitivity and long detection time. Alternatively, the use of continuous crystals for the construction of gamma cameras could effectively improve their sensitivity.

In this study, a novel portable radiation sensor based on a monolithic LYSO crystal was designed, simulated, constructed, and assessed. The novel structure of the radiation sensor with no edges enabled a high filling factor and elevated sensitivity. Monte Carlo simulations showed high decoding accuracy and sensitivity. The experimental results using the VA method at a 511 ± 50 keV energy window revealed an excellent average angular resolution of 0.67°. This value was sufficient for the detection of gamma radiation. In sum, the proposed radiation sensor appears promising for the construction of high-performance radiation detectors or systems.

## 2. Materials and Methods

### 2.1. Simulated Gamma Radiation Sensor

#### 2.1.1. Description of Gamma Radiation Sensor

**Figure 1:**
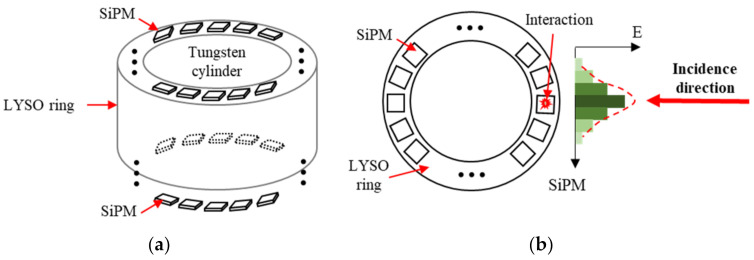
(**a**) Structural diagram of the gamma radiation sensor. (**b**) Illustration of the method used to measure the direction of the incoming gamma ray.

The proposed gamma radiation sensor consisted of three main components: LYSO ring, photosensors, and Tungsten cylinder (Figure 1). LYSO is a wide scintillator used in PET to stop gamma rays. LYSO has a relatively high light yield and is difficult to deliquesce (NaI, CsI, and LaBr_3_ have higher light yields but they are deliquescent). Moreover, LYSO has excellent performance for decay time (Table 1.). Unlike available PET systems, the proposed portable gamma sensor was composed of a single component, a monolithic LYSO crystal ring. This special structure tremendously improved the acceptance angle but required high crystal processing capacity. To detect photons generated from the annihilation of gamma rays and LYSO, the photosensor arrays were coupled to the end face of the LYSO ring. Hence, the direction of incoming gamma rays can be calculated by analyzing the photon distribution. Moreover, a Tungsten cylinder was inserted into the LYSO ring to reject gamma rays penetrating the first LYSO layer.

The incidence direction can accurately be calculated using the distribution of scintillating photons on the detector surface. The positioning accuracy of gamma photons directly determines the decoding resolution of the gamma source.

#### 2.1.2. Implementation of Optical Model in GATE

To validate the feasibility of the design of a continuous LYSO ring, the gamma radiation sensor was simulated in GATE v8.2. The OpticalSystem was selected to model the optical imaging systems for its various boundary processes. The model included two levels: (i) “crystals” and (ii) “pixels”. The “crystals” level consisted of continuous LYSO rings with the inner diameter, outer diameter, thickness, and axial length of the ring set to 48.5, 58.5, 5, and 25.1 mm, respectively (limited by the practical crystal growth). The inner and outer surfaces of the LYSO ring were polished and attached to reflectors. The “pixels” level consisted of two photosensor arrays. Each array was composed of 46 SiPMs evenly distributed on the ring-shaped photosensor array, with the angle and pitch between SiPMs set to 7.83° and 3.7 mm, respectively. A layer of soda-lime glass (0.35 mm in thickness) was set between the light-output surface of the crystal array and upper face of the photodetectors, according to the manual of Sensl J-series SiPM.

The physical processes in the proposed simulation included two components. (1) The electromagnetic processes include photoelectric effect, electron ionization, bremsstrahlung, and positron-electron annihilation. (2) The physical processes include bulk absorption, Mie scattering, processes at boundaries, and wavelength shifting or fluorescence. The properties of the materials used in simulations are shown in Table 2.

Properties of the surface: The UNIFIED surface model was used in GATE. The type, finish, and $\sigma_{\alpha }$ values for each optical interface are presented in Table 3. The crystal surface roughness had little influence on the detector photon propagation in reference [25]. The type of surface between air and reflector was set as dielectric-metal to simulate the packaging of aluminum foil.

#### 2.1.3. Simulation-Based Validation

**Figure 2:**
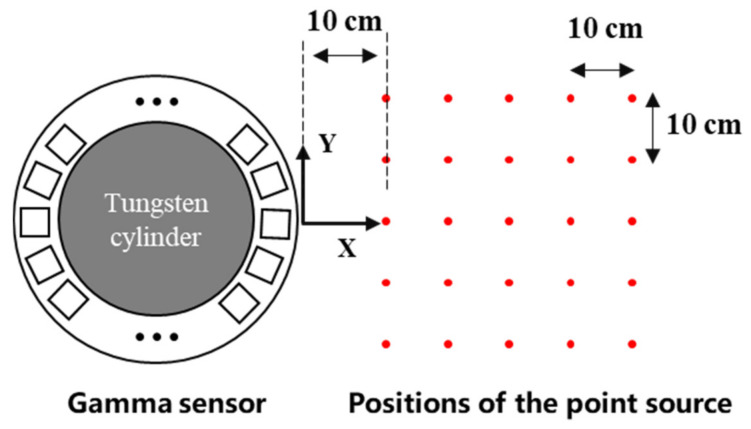
An illustration of the simulation-based experiment. The red points mark the position of the gamma source. The origin of the coordinate is set at a point located on the outer edge of the crystal ring.

In simulations, a coordinate system was established on the central horizontal plane of the monolithic LYSO ring (Figure 2). A spherical source (radius of 0.05 mm, activity of 2.96 MBq, and mono-energy of 511 keV) was used as a source of gamma radiation. To study the relationship between the test position and decoding performance, the source was placed at the 5 × 5 grid points (red in Figure 2). To ensure sufficient amounts of simulation data, the acquisition time of each measurement was set to 10 s. The angular resolution and sensitivity of the system were calculated. The attenuation relationship between attenuation and path length can be described by Equation (1):

(1) $$T=e^{-\mu l}=10^{-\mu 10^{l}}$$

where *T* is the transmittance of the material, *µ* denotes the attenuation coefficient, and *l* refers to the path length of the beam of light through the material sample.

### 2.2. Experimental Validation

#### 2.2.1. Construction of Gamma Radiation Sensor

**Figure 3:**
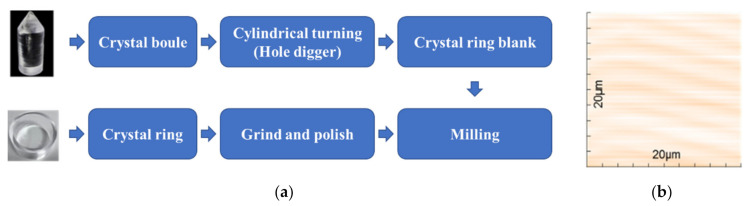
(**a**) The procedures used to fabricate the detector. A hollow crystal ring is daggered from a crystal boule with two diggers of different diameters. The hollow ring is then milled and polished using a polyurethane polishing pad and CeO_2_ polishing solution. (**b**) AFM map of top surface of the ring.

Crystal ring fabrication: The LYSO scintillator boule was grown by the Czochralski method. Moreover, the dimensions of the LYSO boule were normally less than 30 cm in length and 10 cm in diameter. A hollow cylindrical ring (48.5 mm inner diameter, 58.5 mm outer diameter, 5 mm in thickness, and 25.1 mm in length) was fabricated from a LYSO boule using two diggers with different diameters. An ultra-precision surface treatment technology with a polyurethane polishing pad and CeO_2_ polishing solution was employed because LYSO is a hard and brittle material [26]. The procedures to fabricate the monolithic scintillator ring are summarized in Figure 3a. Atomic force microscopy (AFM) was used to measure the roughness of the polished surfaces of the detector ring. As shown in Figure 3b, the surface 3D topography indicated the excellent quality of surface finish. The roughness values (Arithmetical Mean Deviation of Profile, Ra) of the top surface and outer surface were estimated to 1.50 and 2.61 nm, respectively.

Photosensor array ring: To detect the photons generated from the interaction of gamma rays in LYSO, a dual-end readout circuit was coupled with the crystal ring. The readout circuit consisted of two ring-shaped SiPM arrays, on which 46 SiPMs were distributed evenly. Every SiPM (MicroFJ−30035-TSV, Sensl) possessed an active area of 3.16 mm × 3.16 mm and contained 5601 microcells, where each covered an area of 35 μm × 35 μm. LYSO scintillation wavelength (420 nm) and the SiPM peak sensitivity (at 425 nm) matched perfectly. Two SiPM arrays were air-coupled to the two sides of the crystal ring and connected to a high-performance custom-designed electronic module via two 100 mm FPC cables. The electronic module was capable of reading out all the 92 SiPM channels in parallel and then transmitting single event data to a host PC via a USB 2.0 cable. A 1-Bit Sigma-Delta modulation was adapted in the custom-designed 100-channel readout electronics system. Details related to the principles, architectures, and performance can be found in the literature [27,28]. Enhanced specular reflectors (ESR) are ultra-high-reflectivity, mirror-like optical enhancement films. Their nominal reflectance is greater than 98.5%. Two slices of ESR were bent into a circle and attached to the inner and outer circles of the crystal ring, while the outer side of the ESR was secured with adhesive tape.

**Figure 4:**
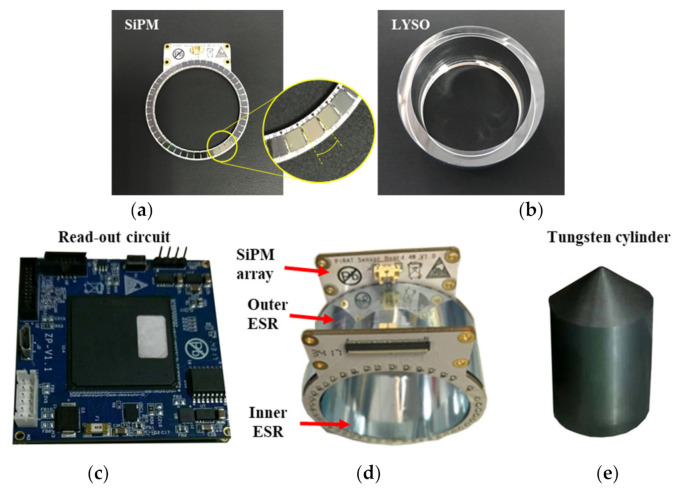
Pictures showing: (**a**) SiPM array ring with 46 SiPMs, (**b**) polished monolithic LYSO scintillator ring, (**c**) custom-designed 100-channel readout electronics system, (**d**) assembled detector module, and (**e**) inserted Tungsten cylinder.

A prototype of the constructed portable gamma radiation sensor is shown in Figure 4.

#### 2.2.2. Experimental Settings

To form a boundary between two layers of crystals for rejecting gamma rays penetrating the first LYSO layer, a Tungsten cylinder was inserted into the center of the crystal ring. As in simulations, a 1.3 MBq ^22^Na point source (0.25 mm in diameter) was placed at 25 locations. The exposure time at each location was 60 s, and the ambient temperature was set to 25 °C throughout the experiments.

### 2.3. Data Analysis

#### 2.3.1. Energy Resolution

**Figure 5:**
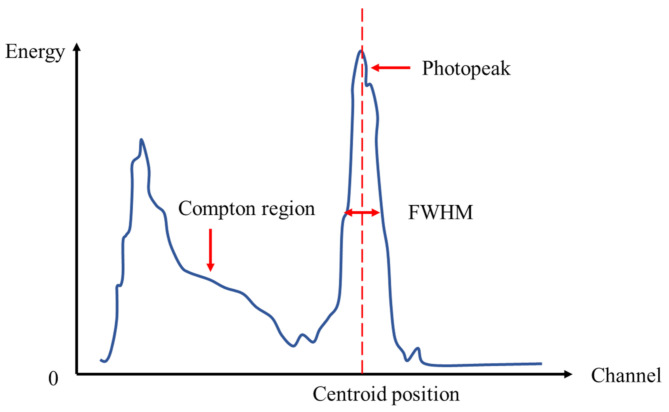
Photon energy spectrum measured by a scintillation detector.

Point sources at 511 keV were used in both simulations and experiments. The total energies of single events were calculated by summing the energy signals from all SiPMs. A typical energy spectrum of the gamma radiation sensor is illustrated in Figure 5. Some incoming 511 keV photons may deposit a portion of their energies and then exit the detector due to the Compton scatter, during which the continuous part of the spectrum (Compton region) was formed. The peak position showed the mean energy of the incoming radiation. The full width at half maximum (FWHM) of the photopeak displayed the effect of fluctuations in the measured charge for the complete deposition of energy by the mono-energetic photons. Therefore, FWHM represented the ability of the sensor to measure the deposited energy. The energy resolution was a dimensionless number and defined as the ratio of photopeak FWHM to its centroid position. To evaluate the impact of gamma source position in both simulations and experiments, the energy resolution and energy spectrum were studied.

#### 2.3.2. Decoding Method

**Figure 6:**
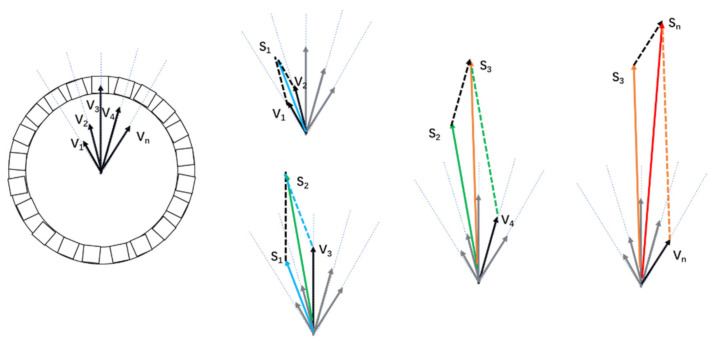
Illustration of the VA method.

Vector addition method (VA method): The energy spectra of all events were first generated, and an energy window was applied to remove the scatters. The average signals of all events by SiPM channels were then calculated as lengths of the vector *V_n_*. N SiPMs near the highest signal were selected as valid signals, where *n* = 9 for one end in this study. Next, the VA method was used to calculate the circumferential direction of the gamma interaction. An illustrative diagram of the VA method is presented in Figure 6. The directions of vectors *V*_1_, *V*_2_, …, *V_n_* were oriented from the center point of the end surface to the central position of SiPMs. The lengths were considered as the readout signals of SiPMs. The vectoral sum *S_n_* of *V*_1_, *V*_2_, …, *V_n_* determined the direction of incoming gamma rays.

Center of gravity method (COG method): Similar to the VA method, the energy spectrum of all events was generated and an energy window was applied to remove the scatters. For every valid event, the SiPMs near the highest signal were selected as valid signals. Afterward, the COG method was used to calculate the circumferential direction of the gamma interaction.

The COG method is a common PET algorithm used to calculate the center of light distribution, as well as locate the interaction of an incoming gamma ray. The location of interaction was calculated using the COG method described by Equation (2):

(2) $$\left\{\begin{array}{c} x_{top}=\frac{\left(E_{t,2}+2*E_{t,3}+\ldots +\left(n-1\right)*E_{t,n}\right)}{E_{t,total}}*360/46\\ x_{bottom}=\frac{\left(E_{b,2}+2*E_{b,3}+\ldots +\left(n-1\right)*E_{b,n}\right)}{E_{b,total}}*360/46 \end{array}\right.$$

where $x_{top}$ and $x_{bottom}$ are the positions of the interaction in the circumferential direction (in degrees). *E_t_*_,2_, *E_t_*_,3_, …, *E_t_*_,*n*_ refer to the energy signals measured by the top circle of SiPMs. *E_b_*_,2_, *E_b_*_,3_, …, *E_b_*_,*n*_ denote the energy signals measured by the bottom circle of SiPMs. $E_{t,total}$ and $E_{b,total}$ are the sum of the energy read by top and bottom SiPMs, respectively. The centers of all distributions for $x_{top}$ and $x_{bottom}$ are calculated as the interaction points. The connection of the interaction point and the center line of the cylinder determines the incoming gamma rays.

#### 2.3.3. Sensitivity of Sensor

The sensitivity is a key index of gamma sensors because it reflects the ability of the system to detect gamma rays. In industrial applications, higher sensitivities mean effective detection of abnormal radiation in the environment. In this study, the sensitivity can be defined according to Equation (3):

(3) $$\eta =n_{detected}/n_{total}$$

where $n_{total}$ is the number of gamma photons that the source emits in total. $n_{detected}$ refers to the number of gamma photons detected by the sensors at the same time.

#### 2.3.4. Impact of Energy Window

Energy windows are often used in quantitative molecular imaging, such as PET and gamma cameras, to filter Compton scatter events. The width of the energy window greatly impacts the image quality and decoding accuracy. Hence, the impacts of energy windows on the accuracy of the proposed gamma radiation sensor were evaluated by implementing different energy windows. The decoding accuracies were calculated at all tested positions in the selected energy windows. The results obtained by VA and COG methods were then compared to determine the best energy window.

## 3. Results and Discussion

### 3.1. Simulation Results and Discussion

#### 3.1.1. Energy Resolution Obtained from Simulations

**Figure 7:**
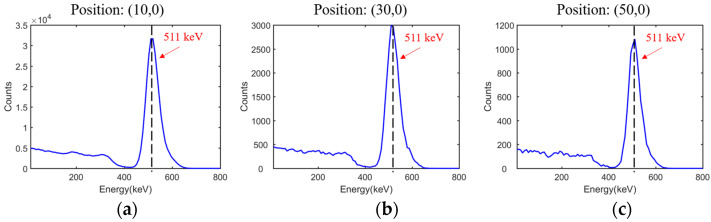
(**a**), (**b**) and (**c**) Simulated energy spectra at positions (10, 0), (30, 0) and (50, 0), respectively.

Typical energy spectra of positions (10, 0), (30, 0) and (50, 0) are displayed in Figure 7, and the energy resolutions at all tested positions are summarized in Table 4. The average and standard errors of energy resolutions were estimated at 11.39% and 0.273%, respectively. For simulation data, no background or intrinsic radiation was detected. The energy resolutions observed with the point source at different positions were almost the same, and the minor differences resulted from the decrease in event number. Meanwhile, the profiles of energy spectra appeared almost the same. Therefore, the impact of the energy window could be neglected.

#### 3.1.2. Angular Resolution Results Obtained from Simulations

**Figure 8:**
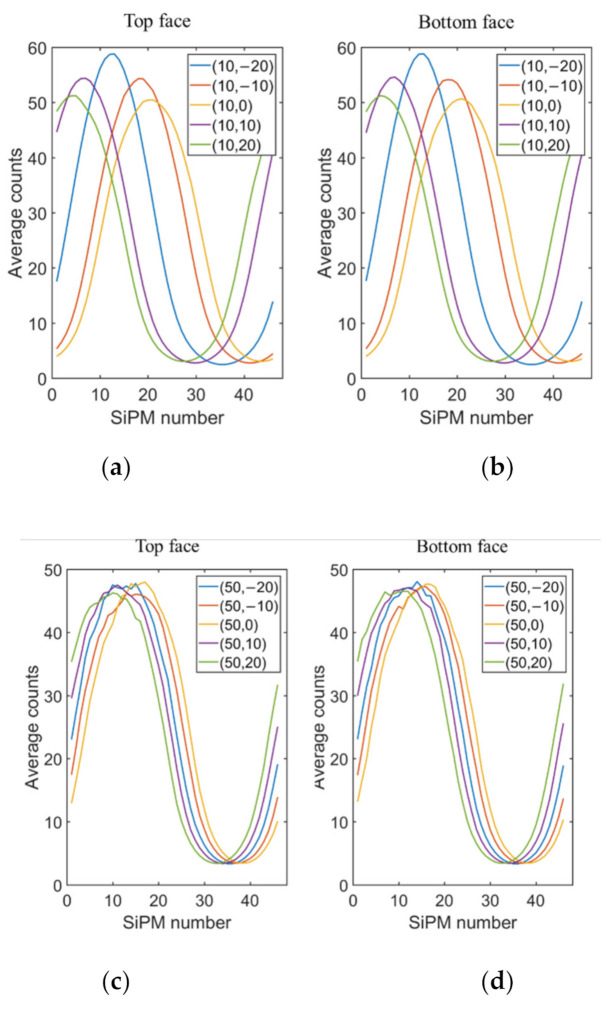
Energy distribution statistics of channels at: (**a**) top face and (**b**) bottom face for ***X*** = 10 cm, and (**c**) top face and (**d**) bottom face for ***X*** = 50 cm.

The average energy distributions by channels at 10 positions (***X*** = 10 cm and ***X*** = 50 cm) are gathered in Figure 8. The peak channels changed obviously with the y coordinate of the test positions (Figure 8a,b), indicating the good resolution of the gamma sensor toward the angular radiation. As the distance changed slightly, the peak energy varied. In Figure 8c,d, the peak channel and peak energy at position ***X*** = 50 cm both showed minor differences since the position of the gamma source changed negligibly when compared to position ***X*** = 10 cm.

**Figure 9:**
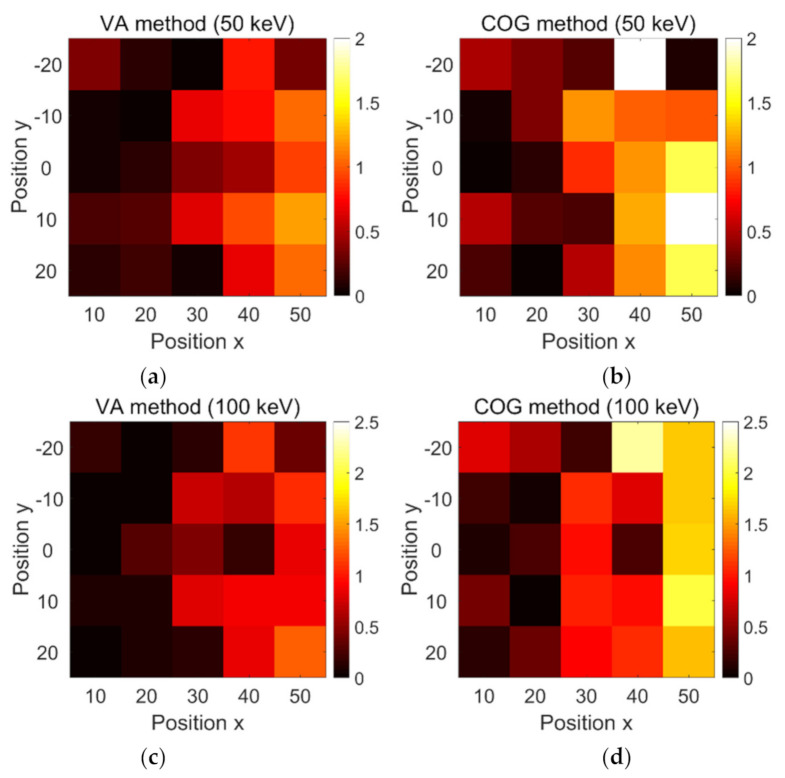
Decoding accuracies of the VA method at (**a**) 50 keV energy window and (**c**) 100 keV energy window, as well as COG method at (**b**) 50 keV energy window and (**d**) 100 keV energy window.

To assess the decoding accuracy of the proposed gamma radiation sensor, an energy window of 511 ± 50 keV was used to filter the Compton scatter events. The VA and COG methods were used to decode the angle of incidence. To better evaluate the decoding errors, the SiPM channels were converted into angular coordinates (in degrees). The decoding accuracies of both methods are summarized in Figure 9. For both methods, the increase in detection distance led to inaccuracy in the angular location from around 0° to 2° for all tested distances. The average angular errors of the VA and COG methods were estimated at 0.452° and 0.738°, respectively. Hence, the VA method showed better accuracy than the COG method.

The influence of the energy window was evaluated at 511 ± 100 keV. Similarly, the angular location became inaccurate for a gamma source far from the sensor. The average angular errors obtained by the VA and COG methods were estimated at 0.446° and 0.846°, respectively. Thus, the VA method showed better decoding performance than the COG method in simulations. On the other hand, the 100 keV energy window showed poor decoding performance when compared to the 50 keV energy window.

#### 3.1.3. Sensitivity Results from Simulation

**Figure 10:**
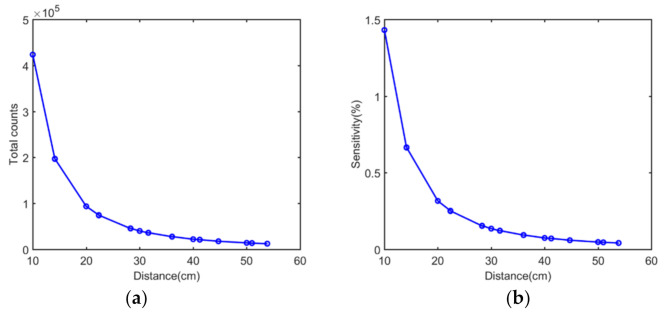
Simulated results of (**a**) events number plot versus distance and (**b**) sensitivity plot versus distance.

The correlations between the total counts of a single simulated acquisition and distance are provided in Figure 10a. The number of detected single photons decreased with the increase in the distance between the sensor and the source. As expected, the curve showed a typical exponential decay distribution. The sensitivity plot shown in Figure 10b agreed well with the event number plot.

### 3.2. Experimental Results and Discussion

#### 3.2.1. Energy Resolution Obtained from Experiments

**Figure 11:**
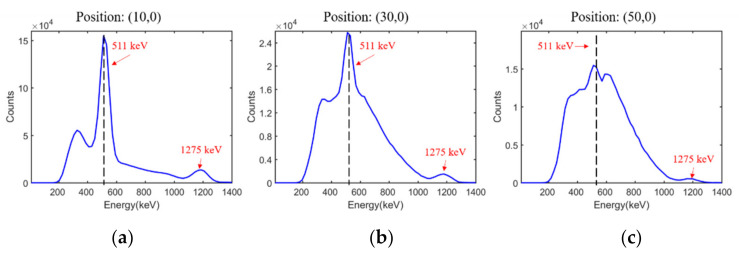
(**a**–**c**) Energy spectra at positions (10, 0), (30, 0), and (50, 0), respectively.

The energy spectra obtained at point sources of (10,0), (30,0), and (50,0) are given in Figure 11. Two peaks at 511 keV and 1275 keV were noticed in all spectra and attributed to ^22^Na. Thus, the proposed detector showed excellent ability to distinguish between different rays with various energies. Moreover, the presence of peaks at 511 keV and 1275 keV demonstrated good readout accuracy, linearity, repeatability, and uniformity in energy measurement.

The energy resolutions at all tested positions are summarized in Table 5. The energy resolutions at positions (10, 0), (30, 0), and (50, 0) were recorded as 14.2%, 64.7%, and 91.1%, respectively. As the distance between the sensor and source increased, the intensity of the radiation from ^22^Na decreased. Unlike in simulations, the proportion of the intrinsic radiation and background radiation increased. This led to a rapid deterioration in energy resolution.

#### 3.2.2. Angular Resolution with Different Energy Windows

**Figure 12:**
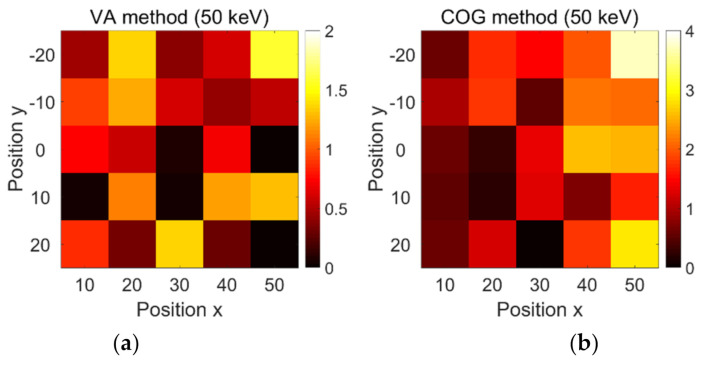

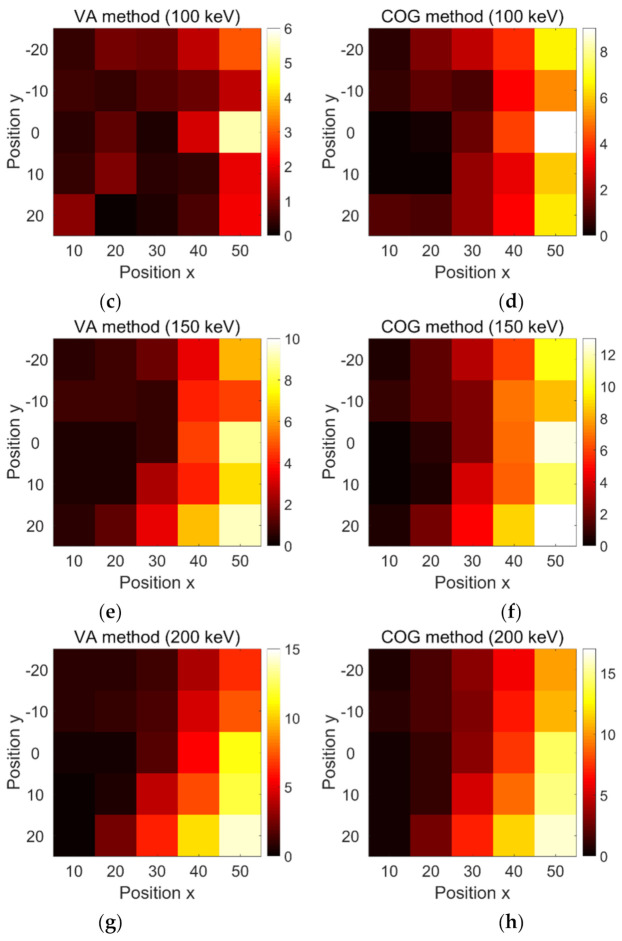
Decoding accuracies obtained by VA method at (**a**) 50 keV, (**c**) 100 keV, (**e**) 150 keV, and (**g**) 200 keV energy window width, as well as using COG method at (**b**) 50 keV, (**d**) 100 keV, (**f**) 150 keV, and (**h**) 200 keV energy window width.

The decoding errors obtained at 25 positions with energy window widths of 50 keV, 100 keV, 150 keV, and 200 keV using the VA and COG methods are shown in Figure 12. Note that SiPM channels were converted into angular coordinates (in degrees).

In Figure 12a, all decoding errors obtained by the VA method with a window width of 50 keV were less than 2°, with an average error value estimated at 0.67°. The performance at different source positions was not regular. The errors at positions (10, −10), (30, 0), (30, −10), (50, 0), and (50, −20) were all close to zero. Furthermore, the declining tendency of the decoding error with distance was not obvious enough.

In Figure 12b, all decoding errors with a window width of 50 keV using the COG method were less than 4°. The average error was estimated at 1.40°. The average decoding errors at positions *X* = 10, 20, 30, 40, and 50 were recorded as 0.64°, 1.04°, 0.93°, 1.83°, and 2.56°, respectively. Hence, the decoding performance obviously became worse with the increase in distance. On the other hand, the VA method showed better decoding performance than the COG method.

The average decoding errors in Figure 12c–h for different combinations of decoding methods and energy windows were estimated to 1.12°, 2.61°, 2.95°, 4.60°, 4.20°, and 5.59°, respectively. Similar conclusions can be drawn from the comparison of Figure 12c,d, Figure 12e,f, and Figure 12g,h. Overall, the VA method showed better decoding performance than the COG method. Moreover, the decoding errors obtained by the VA method deteriorated from around 0.67° to 4.2° when the energy window width increased from 50 keV to 200 keV. Thus, larger energy windows introduced more noise events and worsened the decoding accuracy.

Moreover, all figures (except Figure 12a) indicated that a closer point source led to more accurate positioning.

#### 3.2.3. Sensitivity Results Obtained from Experiments

**Figure 13:**
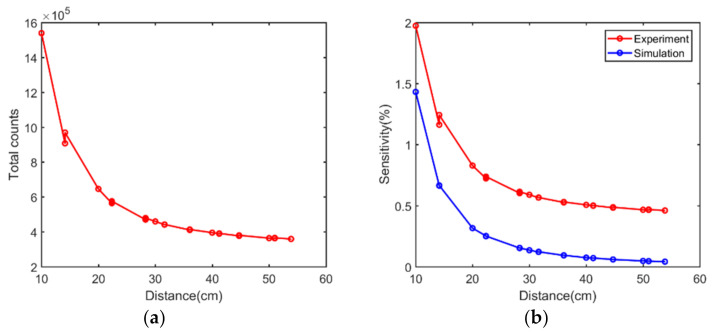
Experimental results of (**a**) events number plot versus distance, and (**b**) sensitivity plot versus distance.

The correlations between the total counts of a single experimental acquisition, sensitivity, and distance are shown in Figure 13a,b. As expected, the curves revealed a typical exponential decay distribution. Overall, the experimental sensitivities were higher than those obtained by simulations due to the presence of background radiation and intrinsic radiation as non-valid events in the detection of gamma rays. This may also explain why larger energy windows introduced more noise events and worsened the decoding accuracy.

## 4. Conclusions

In this study, a novel portable sensor based on a monolithic LYSO ring was successfully developed for the detection of environmental radiation. The sensor was first simulated and then constructed and assessed. The Monte Carlo simulations showed the ability of the sensor to detect gamma rays at 511 keV. In the simulations, a sensor model was constructed, in which the point source with mono-energy of 511 keV was placed at 5 × 5 grid points on the center plane. The simulation data, including the energy resolution, decoding errors, and sensitivity, showed good detection ability of gamma rays. In the experiments, the monolithic LYSO ring was coupled to a custom-designed readout electronic system. A ^22^Na point source was used, and a similar experimental setting to the simulation was carried out. Two decoding methods and different energy windows were implemented to achieve optimal radiation detection. The decoding errors using the VA method at an energy window width of 50 keV were less than 2°, with an average error estimated at 0.67°. This value would suffice for the detection of gamma radiation.

The decoding accuracies of the proposed radiation sensor changed when different energy windows and decoding methods were used. As the energy window increased, the Compton scatter events and noise events interfered with the interaction decoding. The simulation and experimental results both demonstrated the energy window at 511 ± 50 keV as the optical decoding setting. The decoding method also showed a strong impact on the decoding performance. Both simulations and experiments indicated the accuracy of the VA method over the COG method. The optimal average resolution at all tested positions was recorded as 0.67°.

The sensitivity of the proposed radiation sensor was determined by its efficiency and the solid angle. The monolithic gamma sensor has a 100% filling factor and thereby possesses higher detector efficiency than sensors constructed with discrete crystal arrays. Moreover, the proposed radiation sensor displayed great advantages in the detection of radiation from all horizontal directions, different from most gamma sensors, which only detect radiation from one direction. One potential issue of a gamma sensor constructed with a monolithic scintillator ring is the system’s count-rate performance characteristics when it is used in environments with high radiation levels. This issue can be mitigated by reducing the axial length of the MSR detectors.

The proposed method of radiation sensing appears promising for the construction of high-performance radiation detectors or systems. The weight of the proposed radiation sensor, including all the crystals and electrons, was around 200 g and thereby much lighter than most gamma-ray detectors (most weigh over 1 kg [15,21,22]). In this system, larger monolithic LYSO rings can be fabricated to improve the key performance index, such as the sensitivity and decoding performance. Moreover, concise sensing can be carried out by a robot to reach dangerous radiation environments, which is useful for the detection of radiation leakage [29].

However, the proposed radiation sensor based on a monolithic LYSO ring also possessed some potential drawbacks. Firstly, the interaction decoding ability of the proposed sensor was limited in the angular direction. This allowed the calculation of angular interactions instead of accurate interactions. Meanwhile, although the current decoding information was enough for the angular location, the lack of height information will affect its extended use in other image localization methods. Currently, our group is working on a sliced continuous LYSO ring to achieve higher interaction decoding [30]. Moreover, in a complex environment with multiple sources, locating all the sources would be challenging. One possible strategy for detecting all radioactive sources would be through the usage of a network of multiple radiation sensors and a detection system that can promptly and effectively monitor the radiation in nuclear power plants and ports [31].

## Figures and Tables

**Table 1:** The physical parameters of LYSO.

Materials	LYSO (Ce)
Peak Emission (nm)	420
Light Yield (ph/MeV)	30,000–33,000
Density (g/cm^3^)	7.1–7.2
Attenuation @ 1.5 MeV	225.0
Decay Time (ns)	45
Reference	[24]

**Table 2:** Elementary properties of the materials.

Material	Chemical Composition	Density (g·cm^−3^ at 15 ℃)	Refractive Index
Air	N_0.78_O_0.21_Ar_0.01_C_0.00_	1.225 × 10^−3^	1.00
Glass	Na_0.1_Ca_0.05_Si_0.25_O_0.60_	2.5	1.50
Tungsten	W	19.35	-
Reflector	ESR	-	-
Scintillator	Lu_0.71_Y_0.04_Si_0.06_O_0.18_	7.40	1.82

**Table 3:** Type, finish, and σ_𝛼_ value for each optical interface.

Optical Interface	Type	Finish	$\sigma_{\alpha }$
LYSO–Reflector	Dielectric_dielectric	Ground	0.1
LYSO–Glass	Dielectric_dielectric	Polished	0
Glass–SiPM	Dielectric_LUTDAVIS	Detector_LUT	-
Air–Reflector	Dielectric_metal	Ground	0.1

**Table 4:** Energy resolutions at source positions (*X*, *Y*) obtained by simulations.

	*X*(cm)	*X* = 10	*X* = 20	*X* = 30	*X* = 40	*X* = 50
*Y*(cm)						
***Y*** **= −20**		11.45%	11.23%	11.19%	11.18%	11.41%
***Y*** **= −10**		11.78%	11.62%	11.26%	11.30%	10.87%
***Y*** **= 0**		11.69%	11.38%	11.94%	11.55%	11.04%
***Y*** **= 10**		11.64%	11.31%	11.15%	11.13%	11.37%
***Y*** **= 20**		11.36%	11.05%	11.56%	11.22%	11.97%

**Table 5:** Energy resolutions at the source positions (*X*, *Y*) obtained from experiments.

	*X*(cm)	*X* = 10	*X* = 20	*X* = 30	*X* = 40	*X* = 50
*Y*(cm)						
***Y*** **= −20**		21.84%	47.93%	77.79%	87.56%	92.68%
***Y*** **= −10**		16.42%	21.71%	69.70%	83.93%	89.96%
***Y*** **= 0**		14.23%	20.11%	64.69%	82.60%	91.07%
***Y*** **= 10**		18.79%	24.04%	70.96%	84.18%	91.87%
***Y*** **= 20**		25.39%	62.92%	82.07%	87.13%	94.21%

## Data Availability

Not applicable.
